# 

**Published:** 1802-04

**Authors:** 


### To CORRESPONDENTS.

*The great influx of original and temporary matter, which always claims a preference, has obliged us to postpone several valuable Communications as well as books. Drs. Johnstone, Ferguson, Girdlestone, Woodforde, Bellamy, Vage; Mess. Mills, Watkinson, Philo-Medicus, Medicus, A. R. Hare, and Fowler, shall be noticed as soon as possible.*

